# The other *Campylobacters*: Not innocent bystanders in endemic diarrhea and dysentery in children in low-income settings

**DOI:** 10.1371/journal.pntd.0006200

**Published:** 2018-02-07

**Authors:** Ruthly François, Pablo Peñataro Yori, Saba Rouhani, Mery Siguas Salas, Maribel Paredes Olortegui, Dixner Rengifo Trigoso, Nora Pisanic, Rosa Burga, Rina Meza, Graciela Meza Sanchez, Michael J. Gregory, Eric R. Houpt, James A. Platts-Mills, Margaret N. Kosek

**Affiliations:** 1 Department of International Health, Johns Hopkins Bloomberg School of Public Health, Baltimore, United States of America; 2 Biomedical Research, Asociación Benéfica PRISMA, Iquitos, Peru; 3 Department of Environmental Health and Engineering, Johns Hopkins Bloomberg School of Public Health, Baltimore, United States of America; 4 Bacteriology Department, Naval Medical Research Unit-6 (NAMRU-6), Lima, Peru; 5 Facultad de Medicina Humana, Universidad Nacional de la Amazonía Peruana, Iquitos, Peru; 6 Division of Infectious Diseases and International Health, University of Virginia, Charlottesville, United States of America; 7 Division of Infectious Diseases, Johns Hopkins School of Medicine, Baltimore, United States of America; University of California San Diego School of Medicine, UNITED STATES

## Abstract

**Background:**

*Campylobacter* is one of the main causes of gastroenteritis worldwide. Most of the current knowledge about the epidemiology of this food-borne infection concerns two species, *C*. *coli* and *C*. *jejuni*. Recent studies conducted in developing countries and using novel diagnostic techniques have generated evidence of the increasing burden and importance of other *Campylobacter* species, i.e. non-*C*. *coli/jejuni*. We performed a nested case-control study to compare the prevalence of *C*. *coli/jejuni* and other *Campylobacter* in children with clinical dysentery and severe diarrhea as well as without diarrhea to better understand the clinical importance of infections with *Campylobacter* species other than *C*. *coli/jejuni*.

**Methodology/Principal findings:**

Our nested case-control study of 439 stool samples included dysenteric stools, stools collected during severe diarrhea episodes, and asymptomatic stools which were systematically selected to be representative of clinical phenotypes from 9,160 stools collected during a birth cohort study of 201 children followed until two years of age. Other *Campylobacter* accounted for 76.4% of the 216 *Campylobacter* detections by qPCR and were more prevalent than *C*. *coli/jejuni* across all clinical groups. Other *Campylobacter* were also more prevalent than *C*. *coli/jejuni* across all age groups, with older children bearing a higher burden of other *Campylobacter*. Biomarkers of intestinal inflammation and injury (methylene blue, fecal occult test, myeloperoxidase or MPO) showed a strong association with dysentery, but mixed results with infection. MPO levels were generally higher among children infected with *C*. *coli/jejuni*, but *Shigella*-infected children suffering from dysentery recorded the highest levels (26,224 ng/mL); the lowest levels (10,625 ng/mL) were among asymptomatic children infected with other *Campylobacter*. Adjusting for age, sex, and *Shigella* infection, dysentery was significantly associated with *C*. *coli/jejuni* but not with other *Campylobacter*, whereas severe diarrhea was significantly associated with both *C*. *coli/jejuni* and other *Campylobacter*. Compared to asymptomatic children, children suffering from dysentery had a 14.6 odds of *C*. *coli/jejuni* infection (p-value < 0.001, 95% CI 5.5–38.7) but were equally likely to have other *Campylobacter* infections–odds ratio of 1.3 (0.434, 0.7–2.4). Children suffering from severe diarrhea were more likely than asymptomatic children to test positive for both *C*. *coli/jejuni* and other *Campylobacter*–OR of 2.8 (0.034, 1.1–7.1) and 1.9 (0.018, 1.1–3.1), respectively. Compared to the *Campylobacter*-free group, the odds of all diarrhea given *C*. *coli/jejuni* infection and other *Campylobacter* infection were 8.8 (<0.001, 3.0–25.7) and 2.4 (0.002, 1.4–4.2), respectively. Eliminating other *Campylobacter* in this population would eliminate 24.9% of the diarrhea cases, which is almost twice the population attributable fraction of 15.1% due to *C*. *coli/jejuni*.

**Conclusions/Significance:**

Eighty-seven percent of the dysentery and 59.5% of the severe diarrhea samples were positive for *Campylobacter*, *Shigella*, or both, emphasizing the importance of targeting these pathogens to limit the impact of dysentery and severe diarrhea in children. Notably, the higher prevalence of other *Campylobacter* compared to *C*. *coli/jejuni*, their increasing burden during early childhood, and their association with severe diarrhea highlight the importance of these non-*C*. *coli/jejuni Campylobacter* species and suggest a need to clarify their importance in the etiology of clinical disease across different epidemiological contexts.

## Author summary

*Campylobacter* is a major public health concern in developed and developing countries. *C*. *coli* and *C*. *jejuni* have long been considered to be the major disease-causing species, and clinical microbiologic approaches target these two species. However, less selective diagnostic approaches have shown the increasing importance of other *Campylobacter* species (i.e. non-*C*. *coli/jejuni*). Our case-control study investigated the association between diarrhea, *C*. *coli/jejuni*, and other *Campylobacter* among 439 stool samples from 201 children in peri-urban communities in Loreto, Peru. Three quarters of the 216 *Campylobacter* detections were associated with other *Campylobacter*, whose prevalence increased with age and was greater than that of *C*. *coli/jejuni* in all age and clinical groups (dysentery, severe diarrhea, and asymptomatic). Despite their lower prevalence, *C*. *coli/jejuni* were more strongly associated with higher levels of myeloperoxidase, clinical dysentery, and the presence of leukocytes and blood in the stool compared to other *Campylobacter*. Other *Campylobacter* were equally likely as *C*. *coli/jejuni* to be detected in severe diarrhea cases–odds ratio of 1.9 (p-value = 0.018, 95% CI 1.1–3.1) and 2.8 (0.034, 1.1–7.1), respectively. Removing *C*. *coli/jejuni* in this population would eliminate 15.1% of diarrhea compared to 24.9% if other *Campylobacter* were eliminated.

## Introduction

*Campylobacter* are curved gram-negative bacteria and a common cause of enteritis worldwide. The *Campylobacter* genus is composed of 26 species [1], some of which are thermo-tolerant such as *C*. *jejuni* and *C*. *coli*, the most commonly reported *Campylobacter* species affecting humans (82.7% and 17.3%, respectively) [2]. Campylobacteriosis, a food-borne zoonotic infection, is usually diagnosed by selective culture from stool samples, using conditions designed to enhance isolation of *C*. *jejuni* and *C*. *coli* [3,4]. As a result, most of our understanding of the burden and clinical manifestations of campylobacteriosis is specific to *C*. *coli* and *C*. *jejuni*. Diarrhea caused by *Campylobacter* species other than *C*. *coli* and *C*. *jejuni* is thought to be less severe [1], but this common belief is poorly documented [4].

Several studies have identified a significant burden of disease associated with *Campylobacter* infection in children less than five years of age. This includes studies that have demonstrated that *Campylobacter* infection is a risk factor for poor linear growth [5–8]. In the Global Enteric Multicenter Study (GEMS), *Campylobacter* species had the 5^th^ greatest attributable fraction of moderate-to-severe diarrhea in children under 5, with an attributable fraction as high as 16.4% in some areas [9]. The Etiology, Risk Factors and Interactions of Enteric Infections and Malnutrition and the Consequences for Child Health and Development (MAL-ED) is a birth cohort study conducted in eight different countries. Analyses from MAL-ED have also identified a significant burden of diarrhea associated with *Campylobacter* using both enzyme immunoassay (EIA) and polymerase chain reaction (PCR) for detection [6,10]. An analysis of a subset of C*ampylobacter*-positive samples showed that roughly 30% of detections by EIA were due to other *Campylobacter* species, including *C*. *hyointestinalis*, *C*. *troglodytis*, *and C*. *upsaliensis*, which made up an important fraction of the disease burden [6]. This finding is important because other diagnostic tests, both culture and FDA-approved nucleic acid based including tests such as BD Max Enteric Bacterial Panel, BD Diagnostics (Hunt Valley, MD), and BioFire Film Array Gastrointestinal Panel (Salt Lake City, UT) [11], often target *C*. *jejuni* and *C*. *coli* but do not reliably detect other *Campylobacter* species. Prior birth cohort studies in Peru using culture-based methods have also shown that *Campylobacter* is a contributor to the acquisition of postnatal linear growth deficits among children in Peru [5].

Considering the prevalence of *Campylobacter* in diarrhea among children under five years old as identified by EIA in MAL-ED, we performed this nested case-control study using samples from the MAL-ED birth cohort study in Peru to better understand the pathogenicity of the other *Campylobacter* species (i.e. non-*C*. *coli/jejuni* species) among children living in extreme poverty.

## Methods

### Study design and population

We identified all dysentery samples in the Peru MAL-ED cohort at the time of this study (n = 99) and then identified two severe diarrhea and two control/asymptomatic samples matched by the child’s age (± 6 months) using a random number generator. In total, we selected 99 dysentery stool samples, 198 stools from severe diarrhea, and 198 control samples. Diarrhea was defined as maternal report of three or more loose stools in a 24-hour period or at least 1 stool with visible blood present [12], and unique diarrheal episodes were separated by at least 2 days without diarrhea [10]. Dysentery was defined as diarrhea with visible blood reported by the child’s mother during the identified illness episode, and severe diarrhea as diarrhea samples with a modified Vesikari severity score (MAL-ED score) of ≥ 5 [13,14]. Control samples were non-diarrhea stools (i.e. monthly MAL-ED stools). The severe diarrhea samples were selected from a pool of 849 samples, and the control samples from a pool of 8,212 samples which were both ordered and selected by random number generator.

### Ethics statement

The samples used in this study were collected as part of the larger MAL-ED studies following protocols approved by the Institutional Review Board of the Johns Hopkins Bloomberg School of Public Health (Baltimore, USA) and of the Asociación Benéfica PRISMA (Iquitos, Peru). The data analyzed were anonymized to protect the privacy of the participants (S1 Data).

### Stool diagnostic microbiology

Stools were inoculated on *Campylobacter* agar base supplemented with 10% defribrinated sheep’s blood with Blaser’s supplement (Beckton Dickinson, Sparks, MD) containing vancomycin, cephalothin, trimethoprim, polymixin, and amphotericin B. Plates were incubated for 48 hours at 42°C at 5% O_2_, 10% CO_2_, 85% N_2_. If no growth was observed, agar plates were held at least 72 hours to confirm this finding. Colonies with typical morphology were confirmed by gram stain, and oxidase and catalase testing using standard microbiologic techniques. *Campylobacter* colonies were identified as *C*. *jejuni* if they hydrolyzed hippurate.

### Screening for biomarkers of gut inflammation and permeability

Methylene blue was performed as commonly described [15] and fecal occult blood screening was done using commercial cards (Hemoccult, BeckmanCoulter) per manufacturer’s instructions. Samples were also analyzed for myeloperoxidase, MPO, (Alpco, Salem, NH) as described by Kosek *et al*. to assess neutrophil activity [16].

### Culture of *Campylobacter* spp. as positive and negative controls for quantitative PCR (qPCR) analysis

*Campylobacter* and *Shigella* used as controls for qPCR analyses in this study were American Type Culture Collection (ATCC) derived. The lyophilized *C*. *coli* ATCC 33559 was rehydrated in tryptic soy broth and grown under micro-aerophilic conditions (5% carbon dioxide, 10% oxygen and balance nitrogen) for 48 hours at 37°C. A *C*. *jejuni* culti-loop (ATCC 33291) was grown for 10 min with shaking at 37°C in Luria broth then streaked onto blood agar plates with non-selective medium. The lyophilized *C*. *upsaliensis* ATCC 49815 was rehydrated in tryptic soy broth with 5% sheep’s blood and 0.001% pyridoxal-HCl, and cultured on Tryptone Soy Agar (TSA) with 5% sheep’s blood. Both *C*. *jejuni* and *C*. *upsaliensis* were grown under the same above-mentioned micro-aerophilic conditions for 48 hours at 37°C. Given the reported similarity between some *Campylobacter*-associated illness and shigellosis [17,18], we also tested the stools for *Shigella*. *S*. *flexneri* ATCC 12022 2b was grown on TSA Congo Red plates overnight at 37°C.

### qPCR multiplex assays

DNA was extracted from the control bacterial cultures and stools of children using the MO-BIO DNA Power-Soil extraction kit (Carlsbad, CA). Approximately 0.05–0.10 g of feces were weighted and used for DNA extraction. *16S sRNA*, *Campylobacter* adhesin to fibronectin (*cadF*), and invasion plasmid antigen H (ipaH) were used to detect all *Campylobacter* species, *C*. *coli/jejuni*, and *Shigella* by qPCR, respectively. The 25-μl 16S-cadF-ipaH triplex mixtures were prepared using 1 μl of DNA sample, 12.5 μl of Taq Environmental Master Mix 2.0 (ThermoFisher Scientific), 0.375 μl of a primer-probe mix at final concentrations of 0.2 μM for each primer and 0.1 μM for each probe, and nuclease-free water making up the remaining volume. qPCR was performed on a StepOnePlus instrument (Applied Biosystems, Foster City, CA) using the following cycling conditions: 95°C for 10 min, followed by 45 cycles of 95°C for 15 s and 55°C for 1 min.

*C*. *coli* and *C*. *jejuni* were used as positive controls for *cadF*; *C*. *upsaliensis* and *S*. *flexneri* as negative controls for *cadF*. *C*. *coli*, *C*. *jejuni* and *C*. *upsaliensis* were used as positive controls for *16S*, and *S*. *flexneri* as negative control. *S*. *flexneri* served as the positive control for *ipaH*. Given the similar results obtained when we previously tested our positive and negative control samples in triplicate, the stool samples were not run in replicate; however, a subset of samples were run in duplicate for quality control purposes. *C*. *coli*, *C*. *jejuni*, *C*. *upsaliensis* and *S*. *flexneri* controls and negative controls–which included water only (no primers or master mix) and reactions with no DNA template–were included in every run. A cut-off cycle threshold (Ct) of 38 was used to determine positivity. Samples that were *cadF* positive were interpreted as positive for *C*. *jejuni/C*. *coli* (all *cadF* samples were also *16S* positive), samples positive for *Campylobacter 16S* but negative for *cadF* were designated as positive for other *Campylobacter* species. The primers and probes used in this study are listed in Table 1.

**Table 1:** List of primers and probes used for the detection of *Campylobacter* (genus specific), *Campylobacte*r *coli/jejuni*, and *Shigella*.

Detection	Gene	Sequence	Reference
*Campylobacter* genus	*16S rRNA*	**Primer F** 5’- CAC GTG CTA CAA TGG CAT AT -3’ **Primer R** 5’- GGC TTC ATG CTC TCG AGT T -3’ **Probe**^a^ 5’- /56-FAM/CAG AGA ACA /ZEN/ ATC CGA ACT GGG ACA /3IABkFQ/ -3’	[19]
*C*. *coli* and *C*. *jejuni*	*cadF*	**Primer F** 5’- CTG CTA AAC CAT AGA AAT AAA ATT TCT CAC -3’ **Primer R** 5’- CTT TGA AGG TAA TTT AGA TAT GGA TAA TCG -3’ **Probe**^a^ 5’ -/56-JOEN/CAT TTT GAC /ZEN/ GAT TTT TGG CTT GA/3IABkFQ/ -3’	[6]
*S*. *flexneri*	*ipaH*	**Primer F** 5’- CCT TTT CCG CGT TCC TTG A -3’ **Primer R** 5’- CGG AAT CCG GAG GTA TTG C -3’ **Probe** 5’- /56-TAMN/CGC CTT TCC GAT ACC GTC TCT GCA/3IAbRQSp/ -3’	[20]

^a^ A double-quenched probe was used in lieu of BHQ1.

### Statistical analysis

Data analysis was performed using STATA 13.0 (College Station, Texas). Pearson’s chi-squared was used to test the difference between infection status, clinical outcome, age category, and the presence of blood and leukocytes. The difference in MPO levels by infection status and clinical outcome was tested using ANOVA. The odds of *Campylobacter* detection given clinical outcome (control, dysentery and severe diarrhea) were estimated using logistic regression models. We also calculated the odds of experiencing diarrhea (combining both dysentery and severe diarrhea) given infection status using a logistic regression model in order to compute the population attributable fraction (PAF) according to Blackwelder *et al* [21]. Age (broken down into interval of 6 months and treated as a categorical variable), sex, and *Shigella* infections were included as potential confounders based on a priori knowledge [22]. Panel data were analyzed accounting for the clustering at the child level with robust standard errors. Hypothesis testing was done using alpha error of 0.05.

## Results

### Study population characteristics

Of the 99 dysentery stool samples identified in the Peru MAL-ED cohort, 198 severe diarrhea and 198 control samples age-matched to the dysentery samples, 85, 173, and 181 samples were available for testing and analyzed in each group, respectively. These 439 stool samples were from 201 unique children. Approximately 60% of the samples were from male children; the mean age in each clinical group was 12 months (Table 2). Almost all the children aged 0 to 8 months old (n = 145) were breastfed: 96.2%, 98.1%, and 100.0% among the dysentery, severe diarrhea, and control group, respectively. As expected, the difference in sex, age and breastfeeding status among the three clinical groups was not statistically significant (Table 2).

**Table 2:** Study population characteristics by clinical outcome (control, dysentery, and severe diarrhea).

	Control	Dysentery	Severe diarrhea
	(n = 181)	(n = 85)	(n = 173)
**Sex**			
Male	111 (61.3%)	48 (56.5%)	102 (59.0%)
Female	70 (38.7%)	37 (43.5%)	71 (41.0%)
**Age in months, mean (sd)**	11.67 (6.54)	12.44 (6.50)	12.28 (6.33)
**Age group**			
0–6 months	51 (28.2%)	14 (16.5%)	36 (20.8%)
>6–12 months	46 (25.4%)	31 (36.5%)	57 (32.9%)
>12–18 months	57 (31.5%)	23 (27.1%)	46 (26.6%)
>18–32 months	27 (14.9%)	17 (20.0%)	34 (19.7%)
**Breastfeeding status (first 8 months of life)**			
Breastfed	67 (100%)	25 (96%)	51 (98%)
Not breastfed	0 (0%)	1 (4%)	1 (2%)

### Prevalence of *Campylobacter* and *Shigella* infections across clinical group by qPCR

The highest detection of *Campylobacter* was reported in the dysentery group –60 of the 85 samples tested positive–followed by the severe diarrhea (93 of 173) and asymptomatic groups (63 of 181). The prevalence of *C*. *coli/jejuni* was lower than that of other *Campylobacter* across all clinical groups. Among the *Campylobacter*-positive samples, 55.0%, 81.7%, and 88.9% were classified as other *Campylobacter* among the dysentery, severe diarrhea, and control groups, respectively. Overall, 76.4% of all the *Campylobacter* infections (n = 216) were other *Campylobacter* (n = 165).

**Fig 1:**
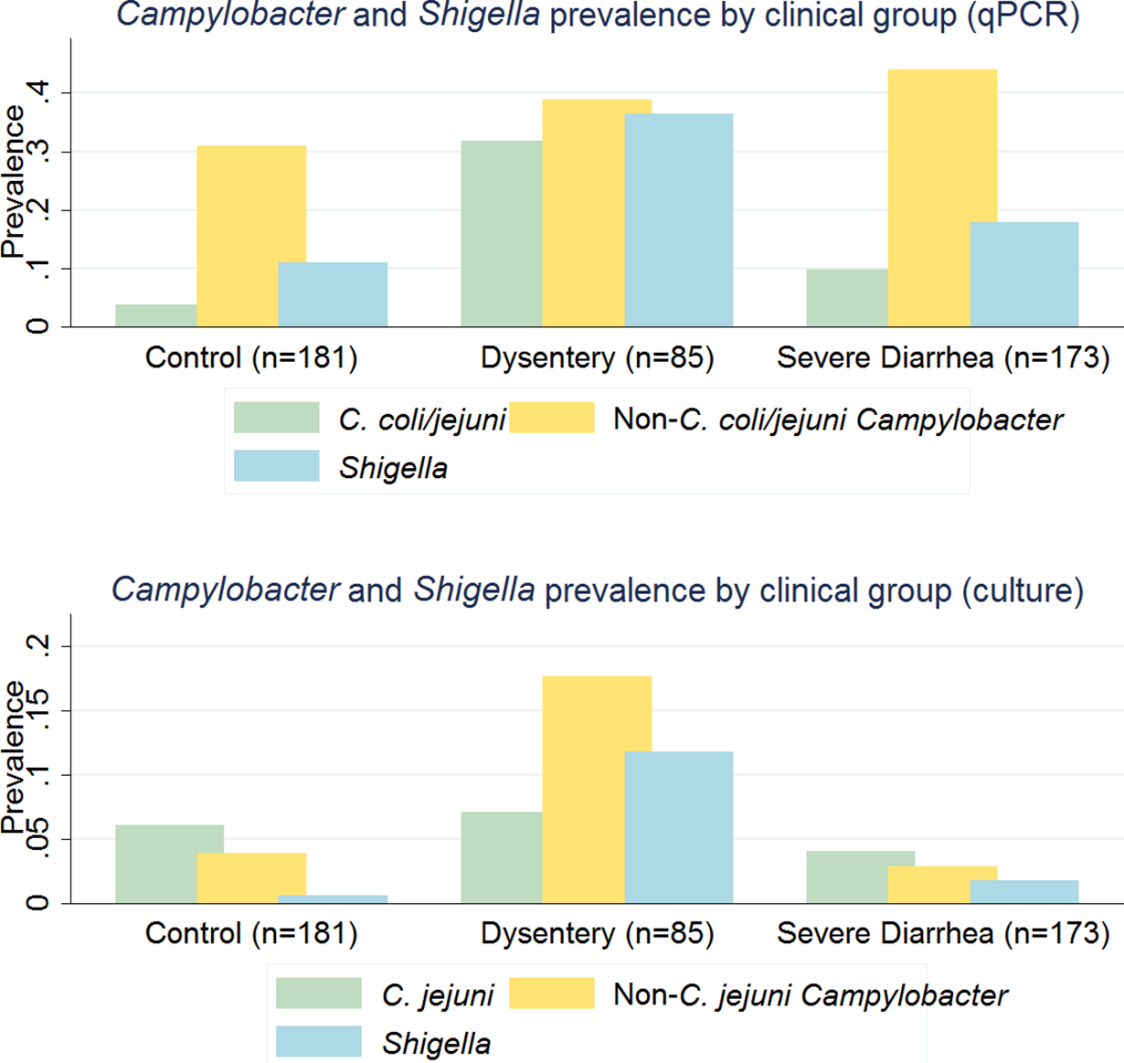
Comparison of *C*. *coli/jejuni*, other *Campylobacter*, and *Shigella* prevalence by clinical group (control, dysentery, severe diarrhea) using qPCR and culture.

*Shigella* infection was also more predominant in the dysentery group where 36.5% of the samples tested positive, compared to 17.9% in the severe diarrhea group and 11.1% in the asymptomatic group. Of the 439 stool samples, 43.1% (n = 189) were negative for infection with *Shigella* and *Campylobacter* while 10.9% (n = 48) were co-infected with both bacteria, most of which were *Shigella* and other *Campylobacter* co-infections. The infection status by clinical group is presented in Table 3. The relatively higher prevalence of other *Campylobacter* was corroborated by the culture-based results performed by our collaborators in Iquitos, Peru (Fig 1).

**Table 3:** *C*. *coli/jejuni*, other *Campylobacter*, and *Shigella* prevalence by clinical outcome (asymptomatic infection, dysentery, severe diarrhea) using the qPCR assay.

	Control (n = 181)	Dysentery (n = 85)	Severe diarrhea (n = 173)	p-value^a^
***C*. *coli/jejuni* (all)**	7 (3.87%)	27 (31.76%)	17 (9.83%)	<0.001*
***C*. *coli/jejuni* & *Shigella***	1 (0.55%)	4 (4.71%)	2 (1.16%)	0.035*
**Other *Campylobacter* (all)**	56 (30.94%)	33 (38.82%)	76 (43.93%)	0.040*
**Other *Campylobacter* & *Shigella***	9 (4.97%)	13 (15.29%)	19 (10.98%)	0.017*
***Shigella***	20 (11.05%)	31 (36.47%)	31 (17.92%)	<0.001*
**Non-*Campylobacter* & non-*Shigella***	108 (59.67%)	11 (12.94%)	70 (40.46%)	<0.001*

^a^ Using Pearson chi-squared

* Statistically significant

qPCR results showed a higher prevalence of other *Campylobacter* (non-*C*. *coli/jejuni*) than *C*. *coli/jejuni* across all clinical groups. It is important to highlight that the culture assay allowed the distinction between *C*. *jejuni* and all other hipppurate negative species of *Campylobacter*, with the latter including *C*. *coli*. The results obtained via culture showed a lower prevalence of all three bacteria (*C*. *jejuni*, other *Campylobacter* (non-*C*. *jejuni*), and *Shigella*) than the qPCR results, notably for the other *Campylobacter*, which is most likely inherent to the lower sensitivity of culture compared to qPCR and the low discriminatory power that culture allows.

### Prevalence of *Campylobacter* and *Shigella* infection across age groups

**Fig 2:**
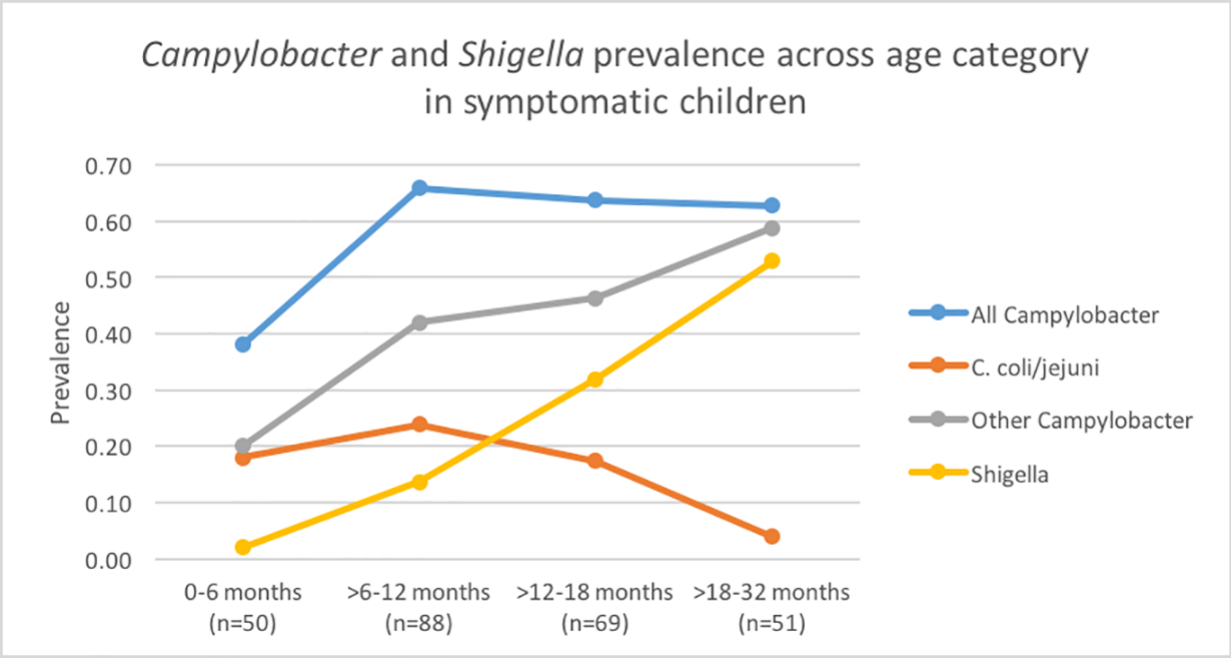
Prevalence of *Campylobacter* and *Shigella* among children suffering from dysentery and severe diarrhea in peri-urban communities in Loreto, Peru.

Children aged 0–6 months were the least burdened by *Campylobacter* or *Shigella* as 29.7% of them tested positive for either bacteria, a lower percentage than the 76.9% observed among children over 18 months old. Across all age groups, the prevalence of other *Campylobacter* was higher than that of *C*. *coli/jejuni* and *Shigella* and followed an overall increasing pattern over time –19.8% among children aged 0–6 months and 52.7% among those over 18 months. *C*. *coli/jejuni* were least common among those over 18 months (2.6%) and most common in the >6-12-month group (19.4%). The pattern of *Shigella* prevalence across age groups mirrored that of other *Campylobacter* with the highest prevalence among those over 18 months (44.9%) and lowest among those 0–6 months (2.0%) (Fig 2).

The prevalence of all *Campylobacter*, other *Campylobacter* (non-*C*. *coli/jejuni*) and *Shigella* increased over time while that of *C*. *coli/jejuni* decreased overall. The isolation frequency for all the bacteria groups increased after 6 months and remained higher than the prevalence observed in the 0–6 month group, except for that of *C*. *coli/jejuni* among children over 18 months old which reached levels less than those among the infants.

### Assessment of intestinal inflammation and permeability based on disease and infection status

To study the association between infection status, clinical outcome, and injury, we performed the fecal occult blood test in conjunction with the methylene blue test (Table 4). Children suffering from dysentery and those infected with *C*. *coli/jejuni* were more likely to have blood present in their stool. The difference across clinical groups for the fecal occult test was only significant among children infected with other *Campylobacter* as 10.7%, 51.5%, and 23.7% of these children tested positive in the control, dysentery, and severe diarrhea group, respectively.

**Table 4:** Biomarkers of mucosal disturbance by clinical outcome (no diarrhea/asymptomatic, dysentery, and severe diarrhea) and infection status (*C*. *coli/jejuni*, other *Campylobacter*, *Shigella*, and non-*Campylobacter* & non-*Shigella*).

	Asymptomatic	Dysentery	Severe diarrhea	p-value
	(n = 181)	(n = 85)	(n = 173)	
***C*. *coli/jejuni***				
**(n = 51)**	**n = 7**	**n = 27**	**n = 17**	
Fecal occult blood (% positive)	14.29%	48.15%	35.29%	0.24^a^
Methylene blue (% positive)	42.86%	51.85%	41.18%	0.77^a^
Myeloperoxidase, ng/mL (sd)*	23,590.77	23,500.15	13,112.29	0.10^b^
	(14,240.89)	(18,334.31)	(10,960.16)	
**Other *Campylobacter***				
**(n = 165)**	**n = 56**	**n = 33**	**n = 76**	
Fecal occult blood (% positive)	10.71%	51.52%	23.68%	<0.05^a^ **
Methylene blue (% positive)	10.71%	39.39%	21.05%	0.01^a^ **
Myeloperoxidase, ng/mL (sd)*	10,624.63	20,279.05	12,469.56	0.01^b^ **
	(13,459.43)	(16,680.93)	(14,528.36)	
***Shigella***				
**(n = 82)**	**n = 20**	**n = 31**	**n = 31**	
Fecal occult blood (% positive)	20.00%	35.48%	22.58%	0.38^a^
Methylene blue (% positive)	10.00%	51.61%	38.71%	0.01^a^ **
Myeloperoxidase, ng/mL (sd)*	12,277.21	26,223.92	17,505.27	<0.05^b^ **
	(10,264.31)	(16,876.25)	(13,484.71)	
**Non-*Campylobacter* & non-*Shigella***				
**(n = 189)**	**n = 108**	**n = 11**	**n = 70**	
Fecal occult blood (% positive)	9.26%	18.18%	12.86%	0.56^a^
Methylene blue (% positive)	10.19%	36.36%	14.29%	0.05^a^ **
Myeloperoxidase, ng/mL (sd)*	12,236.74	17,322.55	10,873.44	0.29^b^
	(10,940.07)	(25,604.9)	(11,689.00)	

^a^ Using Pearson chi-square

^b^ Using ANOVA

* sd = standard deviation

** Statistically significant

A similar trend was observed with the methylene blue test used to detect leukocytes in the stools. Fecal leukocytes were detected primarily in the dysentery group and among children infected with *C*. *coli/jejuni*. Infection with C. *coli/jejuni* was associated with an equal likelihood of children having leukocytes present in their stools regardless of their disease status, which contrasts with other *Campylobacter* infections. Among children who were infected with other *Campylobacter*, 39.4% of those suffering from dysentery had leukocytes present in their stool compared to 10.7% among the asymptomatic children and 21.1% in the severe diarrhea group. As expected, most of the asymptomatic children tested negative for methylene blue whether they were free of any *Campylobacter* and *Shigella*, infected with other *Campylobacter*, or infected with *Shigella* (approximately 10%). This percentage was more than quadrupled when asymptomatic children harbored *C*. *coli/jejuni* infections (42.9%); this difference in percentage was not significant across clinical groups (51.9% and 41.2% in the dysentery and severe diarrhea group, respectively).

A biomarker of environmental enteropathy of interest was myeloperoxidase (MPO). MPO data were available for 415 samples and were missing completely at random (169/181 controls, 80/85 dysentery samples, and 166/173 severe diarrhea samples). As expected, high neutrophil activity correlated with dysentery and *Campylobacter* or *Shigella* infections (Table 4). We aggregated the data presented in Table 4 to compare mean MPO levels across clinical outcome without further breakdown based on infectious status, and mean MPO levels based on infectious status without considering the clinical groups. Dysentery was still associated with the highest MPO levels (23,163 ng/mL), which were significantly different from MPO levels in the control and severe diarrhea groups (12,333 and 11,970 ng/mL, respectively). When we compared the MPO levels between children infected with any *Campylobacter* species and those who were not, the difference was not statistically significant (15,099 and 13,510 ng/mL, respectively). More specifically, *C*. *coli/jejuni* were associated with significantly more inflammation (MPO levels of 19,909 ng/mL) than children who tested negative for *Campylobacter* (13,510 ng/mL) while the difference in MPO levels between children infected with other *Campylobacter* (13,538 ng/mL) and *Campylobacter*-free children was not significant. As expected, the highest MPO levels were noted in children who tested positive for *Shigella* (19,738 ng/mL) and were significantly different from the MPO levels of 13,071 ng/mL measured among those who tested negative.

### Association of *Campylobacter* infection with clinical outcome

The odds of detecting *Campylobacter* in a stool sample given the clinical outcome (dysentery, severe diarrhea, control) were modelled separately for each group of *Campylobacter* species (*C*. *coli/jejuni*, other *Campylobacter*, and all *Campylobacter*) adjusting for age, sex, and *Shigella* infection. Compared to the asymptomatic group, the odds of detecting *C*. *coli/jejuni* were 14.6 among the dysentery samples (p-value <0.001, 95% CI: 5.5–38.7) and 2.8 (p-value = 0.034, 95% CI: 1.1–7.1) among the severe diarrhea samples. Using the same reference group, the odds of being infected with other *Campylobacter* were lower than those of *C*. *coli/jejuni*: children in the dysentery group were equally likely to have other *Campylobacter* in their stools compared to the controls (odds of 1.3, p-value = 0.434, 95% CI: 0.7–2.4) while children in the severe diarrhea group were 1.9 times more likely to have other *Campylobacter* in their stools (p-value = 0.018, 95% CI: 1.1–3.1).

The previous models allowed the comparison of the odds of infection with *C*. *coli/jejuni* (or other *Campylobacter)* in the entire study population (n = 439), i.e. the odds of being infected with *C*. *coli/jejuni* (or other *Campylobacter*) versus not being infected with *C*. *coli/jejuni* (or other *Campylobacter)*, with the latter also including stools that were negative for both *Campylobacter* and *Shigella*. To compare the odds of infection with *C*. *coli/jejuni* to infection with other *Campylobacter*, another logistic regression model was used, restricting the sample size to the stools that were positive for *Campylobacter* spp. (n = 216). Based on this new model, the odds of *C*. *coli/jejuni* infection were 6.6 among the dysentery samples (p-value = 0.006, 95% CI: 1.7–24.9), and 1.8 among the severe diarrhea samples (p-value = 0.298, 95% CI: 0.6–5.7) compared to the controls. This demonstrates that *C*. *coli/jejuni* infections were more likely than other *Campylobacter* infections to be detected among the dysentery samples, but *C*. *coli/jejuni* and other *Campylobacter* were equally likely to be found among severe diarrhea cases.

The overall odds of *Campylobacter* infection (both *C*. *coli/jejuni* and other *Campylobacter* infections) in the study population were significantly increased among the dysentery samples (OR = 4.9, p-value < 0.001, 95% CI: 2.5–9.8) and severe diarrhea samples (OR = 2.4, p-value = 0.001, 95% CI: 1.4–4.0) compared to controls. The odds of *Shigella* infection were also increased in the dysentery samples (OR = 6.4, p-value < 0.001, 95% CI: 2.4–17.3), but the increased odds of 0.9 were not significant in the severe diarrhea samples. There was no significant association between *C*. *coli/jejuni* infection and age, but the increased odds of other *Campylobacter* and *Shigella* infections were statistically significant across age groups, with infections being more prevalent among older children, supporting the results from the descriptive analysis.

### Population attributable fraction (PAF)

To calculate the PAF, the association between diarrhea and infection status was investigated, combining and categorizing the dysentery and severe diarrhea groups as diarrhea (Table 5). Adjusting for sex, age, and *Shigella* infection, children infected with *C*. *coli/jejuni* were 8.8 times more likely to have diarrhea compared to those who tested negative for *Campylobacter* (p-value <0.001, 95% CI: 3.0–25.7). Using the same reference group, infection with other *Campylobacter* increased the odds of diarrhea to 2.4 (p-value = 0.002, 95% CI: 1.4–4.2). In other words, children with *C*. *coli/jejuni*-positive stools were approximately three times more likely to have diarrhea than children infected with other *Campylobacter* species. Adjusting for sex, age, and *Campylobacter* infection, children infected with *Shigella* were approximately three times more likely to have diarrhea (OR = 3.3, p-value = 0.003, 95% CI: 1.5–7.3).

**Table 5:** Association of diarrhea with the detection of *Campylobacter* among children under five years old living in low-income peri-urban settings in Loreto, Peru.

	Odds Ratio	p-value	95% CI
***Campylobacter***			
*Campylobacter negative*	Reference		
*C*. *coli/jejuni*	8.82	<0.001*	3.03–25.68
Other *Campylobacter*	2.42	0.002	1.40–4.22
***Shigella***	3.29	0.003	1.48–7.29
**Sex**			
Male	Reference		
Female	1.16	0.598	0.66–2.03
**Age group (in months)**			
0–6	Reference		
>6–12	1.46	0.270	0.75–2.85
>12–18	0.79	0.537	0.37–1.67
>18–32	1.15	0.750	0.48–2.77

Adjusting for gender, and age and infection with *Shigella*, the odds of having diarrhea is greater in children with *C*. *coli/jejuni* than other *Campylobacter* species, but other *Campylobacter* species are still associated with diarrhea. When their increased prevalence is accounted for they have a higher attributable fraction of diarrhea than *C*. *coli/jejuni*.

* Statistically significant

With other *Campylobacter* detected in 42.3% of the diarrhea samples and odds of diarrhea of 2.4, approximately a quarter of all diarrhea cases in this study (24.9%, 95% CI: 12.1–32.2%) was attributed to other *Campylobacter*; this PAF was higher than that of *C*. *coli/jejuni*. Indeed, eliminating *C*. *coli/jejuni* would only eliminate 15.1% of the diarrhea cases (95% CI: 11.4–16.4%) considering their low prevalence among the diarrhea samples (0.2), despite the odds of diarrhea of 8.8. Overall, 40.8% of the diarrhea cases (95% CI: 27.1–48.6%) could be attributed to *Campylobacter* (prevalence of 0.6), which is more than double the PAF of 16.7% (95% CI: 7.8–20.7%) due to *Shigella* (prevalence of 0.2).

## Discussion

Our study of the epidemiology of *Campylobacter* among children in peri-urban communities in the Peruvian Amazon underscores the importance of other *Campylobacter* species (i.e. non-*C*. *coli/jejuni*) in the etiology of diarrhea. Other *Campylobacter* had the highest attributable fraction for childhood diarrhea (24.9%) and accounted for more infections than *C*. *coli/jejuni* in all three clinical groups (asymptomatic, dysentery, and severe diarrhea), comprising more than half of all *Campylobacter* infections (165 of the 216 *Campylobacter* detections). Although *C*. *coli/jejuni* were more strongly associated with dysentery, other *Campylobacter* were equally likely as *C*. *coli/jejuni* to be detected in severe diarrhea cases. The lower percentage of other *Campylobacter* detected using culture methods is most likely due to the lower sensitivity of this diagnostic tool optimized for the detection of *C*. *jejuni*, and the higher sensitivity of qPCR compared to culture [4,23]. The higher prevalence of other *Campylobacter* compared to that of *C*. *coli/jejuni* is at odds with some of the published literature which has shown that *C*. *coli/jejuni* are responsible for more than 80% of all *Campylobacter* infections and are the only species of major public health importance [2,3]. However, it is important to highlight that the prevalence of *C*. *coli/jejuni* does fall within the reported range in developing countries [4,24], suggesting that the discrepancy between our results and published literature stems from the fact that we were detecting more of the non-*C*. *coli/jejuni* species [3]. Additionally, others have also reported instances where more than 50% of the *Campylobacter* isolated were non-*C*. *coli/jejuni* species [25]. As more research is being conducted in developing countries using diagnostic techniques that detect other *Campylobacter* species more reliably, the importance of non-*C*. *coli/jejuni* species has been raised [3,4,25,26]. Discordance analysis of BioFire rapid diagnostic system also reported a high amount of culture-negative but probe-positive specimens in a study conducted in different clinics across the USA (58 positive using BioFire compared to 35 positive using culture) [11], suggesting the other *Campylobacter* may not be entirely restricted to impoverished settings.

Our study also found evidence that the detection of other *Campylobacter* was more frequent than *C*. *coli/jejuni* among children across all age groups and increased with age while that of *C*. *coli/jejuni* decreased in the second year of life. Despite the overall increase in diarrhea and infection with all *Campylobacter*, other *Campylobacter*, and *Shigella* from 0 to 18 months, the illness-to-infection ratio decreased over time during the first 18 months. This trend, not observed with *C*. *coli/jejuni*, suggests a shift towards more asymptomatic infections which may be explained by the natural immunity developed after early exposure to these bacteria [3,17,24]. Why such trend is not observed with *C*. *coli/jejuni* remains unclear and requires additional investigation. The surge in the illness-to-infection ratio observed among the children aged 18–32 months might be due to the relatively small number of children in that age group, and requires additional investigation with a larger study population to validate these findings.

Some of our results about the association between MPO and clinical outcome were unexpected. Since *Shigella* and *Campylobacter* are known pathogens that cause inflammatory diarrhea [18], a positive association was expected between the level of MPO and infection with any of these pathogens. Our finding of no significant association between MPO and infection with any *Campylobacter* species is at odds with McCormick *et al*.’s study that reported a consistent and positive association between MPO and *Campylobacter* which had data from all eight MAL-ED sites and greater power to detect associations [7,27]. However, it is important to highlight that we did observe a significant positive association between MPO and *C*. *coli/jejuni* compared to the negative-*Campylobacter* group. Despite this positive association, our overall results of no association between MPO and infection with any *Campylobacter* species seem to be driven by the lack of significant association between MPO and other *Campylobacter*, which accounted for most of the *Campylobacter* infections.

A limitation of our study was a relatively low number of dysentery and severe diarrhea cases, as this study was a community-based study, and most episodes in such cases are mild to moderate. Another limitation was the focus on only two pathogens, *Campylobacter* and *Shigella*. Other microbes most likely contributed to symptomatic infections, especially in the severe diarrhea group. Other diarrhea-causing agents that could be added to the assay include enterohemorrhagic *E*. *coli* and *Salmonella*, as well as *Yersinia* and *E*. *histolytica* as alternate causes of bloody and severe diarrhea [28]. These pathogens would not be expected to be very prevalent relative to *Shigella* and *Campylobacter* and indeed have been evaluated in this population (10). However, this more inclusive assay would be useful for the evaluation and treatment of individual patients in other epidemiologic settings. Of note, enteroinvasive *E*. *coli* would have been classified as *Shigella* with our PCR assay and is implicitly contained in the data presented. Despite these limitations, our study showed a clear association between clinical outcome and specific *Campylobacter* species. This finding is consistent with other studies that reported that the highest burden of diarrhea in Loreto, Peru and Venda, South Africa was associated with *Campylobacter* in the first year of life, and that *Campylobacter* is the most frequently isolated pathogen in Loreto, Peru [4]. The very few *C*. *coli/jejuni* infections detected among children aged 18–32 months (n = 2) might be an artifact of the rather small number of samples from children in that age group. Most importantly, the burden of other *Campylobacter* was most likely underestimated in our study which defined other *Campylobacter* as positive for *16S rRNA* but negative for *cadF*. Since infections with *C*. *coli/jejuni* and other *Campylobacter* are not mutually exclusive, it is possible that some of the *C*. *coli/jejuni*-positive samples were also positive for other *Campylobacter*. A comparison of the *cadF* and *16S rRNA* Ct values in the *C*. *coli/jejuni* group revealed that 50 out of the 51 samples had a Ct difference greater than 8. Given the average three copies of *16S rRNA* per *C*. *coli* and *C*. *jejuni* genome [29–31], the burden and impact of other *Campylobacter* is most likely greater than reported. A more refined assessment of the burden of these other *Campylobacter* species will require sequencing or the development of assays that detect gene sequences specific to these species, which would strengthen our findings. Lastly, the importance of co-infections and how to attribute disease burden in this and other studies of enteric disease in the setting of comprehensive diagnostics remains a challenge, but the random selection of samples from the collection makes alternative co-infections unlikely to undermine study findings.

Overall, our study highlights the importance of other *Campylobacter* in the etiology of diarrheal disease and contributes to filling the knowledge gap about the epidemiology of *Campylobacter* infection in developing countries. Although *C*. *coli/jejuni* are important causes of dysentery, the severity of disease caused by non-*C*. *coli/jejuni Campylobacter* species and the frequency with which they are detected combined with a greater than expected association with moderate to severe diarrhea strongly suggest that they are the cause of an important fraction of the *Campylobacter* burden in children in the developing world. Further work is underway to determine which of these other *Campylobacter* species are most prevalent and strongly associated with both clinical disease, enteropathy and the acquisition of linear growth deficits in this population.

## Supporting information

S1 Checklist Strobe checklist. (DOC)

S1 Data Data used for this paper. (CSV)
