# Supplementary material for: The other Campylobacters: Not innocent bystanders in endemic diarrhea and dysentery in children in low-income settings
Source: PLoS Negl Trop Dis. 2018 Feb 7;12(2):e0006200. doi: 10.1371/journal.pntd.0006200 (PMC5819825; doi:10.1371/journal.pntd.0006200)
Supplement: S1 Checklist — (DOC) [file pntd.0006200.s001.doc]

STROBE Statement—Checklist of items that should be included in reports of ***case-control studies***

|  | Item No | Recommendation |
| --- | --- | --- |
| **Title and abstract** | 1 | (*a*) Indicate the study’s design with a commonly used term in the title or the abstract  **Page 1** |
|  |  | (*b*) Provide in the abstract an informative and balanced summary of what was done and what was found  **Pages 2 and 3** |
| Introduction | | |
| Background/rationale | 2 | Explain the scientific background and rationale for the investigation being reported  **Lines 71 to 94** |
| Objectives | 3 | State specific objectives, including any prespecified hypotheses  **lines 95 to 98** |
| Methods | | |
| Study design | 4 | Present key elements of study design early in the paper  **Lines 101 to 116** |
| Setting | 5 | Describe the setting, locations, and relevant dates, including periods of recruitment, exposure, follow-up, and data collection  **Lines 101 to 116** |
| Participants | 6 | (*a*) Give the eligibility criteria, and the sources and methods of case ascertainment and control selection. Give the rationale for the choice of cases and controls  **Lines 101 to 116** |
|  |  | (*b*) For matched studies, give matching criteria and the number of controls per case  **Lines 101 to 116** |
| Variables | 7 | Clearly define all outcomes, exposures, predictors, potential confounders, and effect modifiers. Give diagnostic criteria, if applicable  **Lines 117 to 160** |
| Data sources/ measurement | 8* | For each variable of interest, give sources of data and details of methods of assessment (measurement). Describe comparability of assessment methods if there is more than one group  **Lines 117 to 116** |
| Bias | 9 | Describe any efforts to address potential sources of bias  **N/A (Lines 117 to 116)** |
| Study size | 10 | Explain how the study size was arrived at  **Lines 101 to 116** |
| Quantitative variables | 11 | Explain how quantitative variables were handled in the analyses. If applicable, describe which groupings were chosen and why  **Lines 101 to 171** |
| Statistical methods | 12 | (*a*) Describe all statistical methods, including those used to control for confounding  **Lines 161 to 171** |
|  |  | (*b*) Describe any methods used to examine subgroups and interactions  **Not applicable** |
|  |  | (*c*) Explain how missing data were addressed  **Not applicable** |
|  |  | (*d*) If applicable, explain how matching of cases and controls was addressed  **Lines 161 to 171** |
|  |  | (*e*) Describe any sensitivity analyses  **Not applicable** |
| Results | | |
| Participants | 13* | (a) Report numbers of individuals at each stage of study—eg numbers potentially eligible, examined for eligibility, confirmed eligible, included in the study, completing follow-up, and analysed  **Lines 174 to 177** |
|  |  | (b) Give reasons for non-participation at each stage  **Not applicable** |
|  |  | (c) Consider use of a flow diagram  **Not applicable** |
| Descriptive data | 14* | (a) Give characteristics of study participants (eg demographic, clinical, social) and information on exposures and potential confounders  **Lines 174 to 177** |
|  |  | (b) Indicate number of participants with missing data for each variable of interest  **Lines 174 to 177 and 241 to 243** |
| Outcome data | 15* | Report numbers in each exposure category, or summary measures of exposure  **Lines 184 to 262** |
| Main results | 16 | (*a*) Give unadjusted estimates and, if applicable, confounder-adjusted estimates and their precision (eg, 95% confidence interval). Make clear which confounders were adjusted for and why they were included  **Lines 264 to 312** |
|  |  | (*b*) Report category boundaries when continuous variables were categorized  **Lines 264 to 312 and 161 to 171** |
|  |  | (*c*) If relevant, consider translating estimates of relative risk into absolute risk for a meaningful time period  **Not applicable** |

| Other analyses | 17 | Report other analyses done—eg analyses of subgroups and interactions, and sensitivity analyses  **Not applicable** |
| --- | --- | --- |
| Discussion | | |
| Key results | 18 | Summarise key results with reference to study objectives  **Lines 315 to 353** |
| Limitations | 19 | Discuss limitations of the study, taking into account sources of potential bias or imprecision. Discuss both direction and magnitude of any potential bias  **Lines 354 to 375** |
| Interpretation | 20 | Give a cautious overall interpretation of results considering objectives, limitations, multiplicity of analyses, results from similar studies, and other relevant evidence  **Lines 315 to 375** |
| Generalisability | 21 | Discuss the generalisability (external validity) of the study results  **Lines 376 to 381** |
| Other information | | |
| Funding | 22 | Give the source of funding and the role of the funders for the present study and, if applicable, for the original study on which the present article is based  **Lines 388 to 394** |

*Give information separately for cases and controls.

**Note:** An Explanation and Elaboration article discusses each checklist item and gives methodological background and published examples of transparent reporting. The STROBE checklist is best used in conjunction with this article (freely available on the Web sites of PLoS Medicine at http://www.plosmedicine.org/, Annals of Internal Medicine at http://www.annals.org/, and Epidemiology at http://www.epidem.com/). Information on the STROBE Initiative is available at http://www.strobe-statement.org.
